# Remimazolam Anesthesia for MitraClip Implantation in a Patient with Advanced Heart Failure

**DOI:** 10.1155/2021/5536442

**Published:** 2021-05-05

**Authors:** Tomoe Satoh, Noriaki Nishihara, Yasuaki Sawashita, Sho Ohno, Naoyuki Hirata, Michiaki Yamakage

**Affiliations:** Department of Anesthesiology, Sapporo Medical University School of Medicine, South 1, West 16, Chuo-ku, Sapporo, Hokkaido 060-8543, Japan

## Abstract

Remimazolam, a novel and ultrashort-acting benzodiazepine, has been available for general anesthesia in Japan. The administration of remimazolam does not induce injection pain, has been reported to have less cardiovascular depressant effects during general anesthesia, and flumazenil can antagonize the effects of remimazolam. However, in clinical trials, no patient who is complicated with severe heart failure or undergoes cardiac surgery was included. We present anesthetic management with remimazolam for MitraClip^®^ implantation in a patient with severe mitral regurgitation and advanced heart failure. Remimazolam was administered both in anesthetic induction and maintenance with less cardiovascular depressant effects. After surgical procedures were completed, the patient smoothly recovered from anesthesia and the tracheal was extubated just after administration of flumazenil. Remimazolam may be able to achieve appropriate anesthetic management in patients complicated with severe cardiovascular diseases.

## 1. Introduction

Since existing intravenous and volatile anesthetics have cardiovascular depressant effects, they should be administered with discretion to avoid critical hypotension and bradycardia in patients with severe heart failure. Particularly in cardiac surgery, the methods of anesthetic induction and maintenance for such patients may depend on personal experience and/or institutional policy.

Remimazolam, a novel and ultrashort-acting benzodiazepine, is available for general anesthesia in Japan. The administration of remimazolam does not induce injection pain, has been reported to have less cardiovascular depressant effects during general anesthesia, and its effects can be reversed by flumazenil [1, 2]. Therefore, remimazolam has been expected to achieve more appropriate induction and recovery in general anesthesia compared to existing intravenous and volatile anesthetics. However, in clinical trials, no patient with severe heart failure or who was undergoing cardiac surgery was included [1, 2]. We present a case of general anesthetic management with remimazolam for MitraClip implantation in a patient with advanced heart failure.

## 2. Case Presentation

A 79-year-old woman had chronic heart failure (New York Heart Association functional class III), with ischemic heart disease that had been treated by coronary artery bypass grafting 20 years earlier, and multiple percutaneous coronary interventions had been performed. Eight years earlier, heart failure with a low ejection fraction (<35%) had developed, and she then underwent cardiac resynchronization therapy for compromised electrical activity. While she had been repeatedly hospitalized for heart failure, mitral regurgitation (MR) developed. Transthoracic echocardiography (TTE) showed severe MR with a very low ejection fraction of 33% and diffuse severe hypokinesis. Since the surgical risk for mitral valve replacement was quite high, with a Society of Thoracic Surgery (STS) score of 19.9% and a European System for Cardiac Operative Risk Evaluation (EuroSCORE II) of 21.9%, MitraClip (Abbott Vascular, Chicago, IL, USA) implantation was scheduled [3].

**Figure 1:**
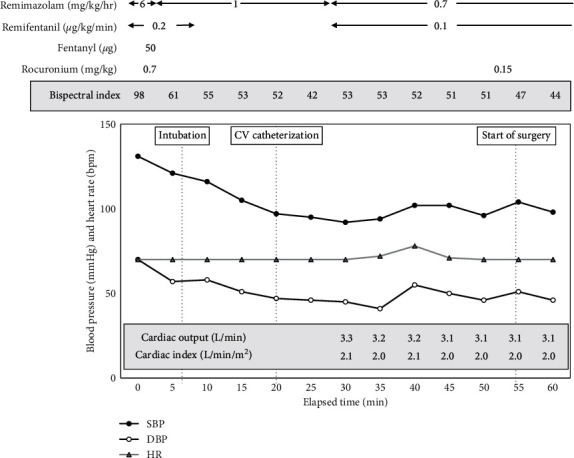
The anesthetic chart from anesthetic induction to the start of surgery. Any vasopressor agent was not used to maintain hemodynamics during the period of anesthetic induction.

Anesthesia was induced with remimazolam, remifentanil (0.20 *μ*g/kg/min), and rocuronium (0.7 mg/kg). Remimazolam was administered at 6 mg/kg/h until loss of consciousness, followed by 1 mg/kg/h of remimazolam for maintenance. Time to loss of consciousness was about 2 minutes after the start of remimazolam administration. The patient was intubated 3 min after rocuronium administration of 0.7 mg/kg. Monitoring followed a routine protocol for mitral clip implantation that included the use of transesophageal echocardiography and the FloTrac monitoring system (Edwards Lifesciences, Irvine, CA, USA), which can measure continuous cardiac output, cardiac index, and stroke volume.  Figure 1 shows the anesthetic chart from anesthetic induction to the start of surgery. During the period from anesthetic induction to the start of surgery, no vasopressor agent was used to maintain hemodynamics. The dose of remimazolam was adjusted to a bispectral index range from 40 to 60, and it could be decreased by 0.15 mg/kg/h during surgical procedures. MitraClip implantation via a femoral vein approach was successfully performed without any complications. After firm pressure was applied for 15 minutes to treat bleeding at a catheter insertion site, remimazolam was discontinued, and 0.2 mg of flumazenil was administered. About three minutes after the administration of flumazenil, the patient's eyes opened and she breathed spontaneously. The tracheal tube was removed. After extubation, there were no adverse events, including resedation or desaturation.

## 3. Discussion

Successful general anesthesia management with remimazolam for MitraClip implantation in a patient with advanced heart failure was reported. Remimazolam has been available for general anesthesia since August 2020 in Japan. However, previous clinical trials of remimazolam for general anesthesia have not been conducted in patients with severe heart failure or who were undergoing cardiac surgery [1, 2]. This is the first case of anesthetic management with remimazolam for MitraClip implantation in a patient with severe heart failure.

While the dose of 12 mg/kg/h of remimazolam is generally recommended for anesthetic induction on the basis of clinical trials, a dose of 6 mg/kg/h was used with discretion because of heart failure. Although the dose administered was lower than the recommended dose, the patient was smoothly anesthetized, and the time to loss of consciousness was about 2 minutes. A remimazolam dose of 1 mg/kg/h is recommended for anesthetic maintenance. In the present case, the maintenance dose was decreased by 0.15 mg/kg/h on the basis of the bispectral index (range from 40 to 60). It has been demonstrated that cardiac output affects the hypnotic dose of intravenous anesthetic agents via modulation of drug metabolism [4, 5]. As the cardiac output decreased, the hypnotic dose of propofol and the time to achieve anesthesia decreased [5]. Although the effects of cardiac output on the blood concentration of remimazolam have not been fully elucidated in subjects with heart failure, the low cardiac output (<4.0 L/min) of the present patient might have allowed the reduction in the dose of remimazolam. Accumulated clinical evidence and experience may propose a more appropriate dose of remimazolam for general anesthesia on the basis of a patient's clinical status.

Previous studies have reported perioperative hypotension, which is associated with postoperative myocardial injury and adverse events [6–9]. The incidence of perioperative hypotension during the period of anesthetic induction has been reported to be more than that during the period of surgical procedures [10, 11]. A recent study reported that even short-term hypotension during anesthetic induction could induce postoperative renal injury [11]. Thus, the preservation of hemodynamics during anesthetic management is desired to improve postoperative outcomes. Particularly in patients with cardiovascular complications, anesthesiologists should administer anesthetic agents carefully to avoid cardiovascular depression. For such populations, the methods and agents for general anesthesia may be applied variously depending on the anesthesiologists' experience and institutional policy [12]. While the dose of sedative agents is decreased to minimize the effects of agent on hemodynamics, anesthesiologists require scrupulous attention to intraoperative awareness [13, 14]. In the present case, the protocol for remimazolam administration did not induce cardiovascular depression or intraoperative awareness. Clinical trials of remimazolam for general anesthesia have shown that the incidence of hypotension in patients anesthetized with remimazolam was lower than with propofol in noncardiac surgery [1]. A previous meeting report on clinical trials in Germany showed that the use of vasopressors was significantly lower in patients receiving remimazolam at 6 mg/kg/h than in patients on propofol and sevoflurane in all phases of cardiac surgery [15]. Though a randomized clinical trial is required to evaluate the cardiovascular depressant effects of remimazolam compared to existing anesthetics, remimazolam may be able to be used safely with less cardiovascular depressant effects in high-risk patients.

In cardiac surgery, it has been well known that volatile, but not intravenous, anesthetics (propofol and midazolam) have cardioprotective effects [16, 17]. When intravenous anesthetics are administered with volatile anesthetics, there may be interference with the cardioprotective effects of volatile anesthetics [18]. The cardioprotective effects of remimazolam and interactions between remimazolam and volatile anesthetics have not been elucidated. Future studies are needed to address these issues.

## Data Availability

The data that support the findings of this study are available from the corresponding author, N.H., upon reasonable request.
